# Benzothia­zol-2-amine–3-meth­oxy­carbonyl-7-oxabicyclo­[2.2.1]hept-5-ene-2-carb­oxy­lic acid (1/1)

**DOI:** 10.1107/S1600536810052542

**Published:** 2010-12-18

**Authors:** Jian Li

**Affiliations:** a Department of Chemistry and Chemical Engineering, Weifang University, Weifang 261061, People’s Republic of China

## Abstract

In the title 1:1 adduct, C_7_H_6_N_2_S·C_9_H_10_O_5_, all non-H atoms of the benzothia­zol-2-amine mol­ecule are essentially coplanar, with a maximum deviation of 0.0286 (9) Å for the S atom. In the crystal, inter­molecular N—H⋯O and O—H⋯N hydrogen bonds connect two mol­ecules of each type into centrosymmetric four-component clusters.

## Related literature

For applications of 3-(meth­oxy­carbon­yl)-7-oxa-bicyclo­[2.2.1]hept-5-ene-2-carb­oxy­lic acid and its derivatives, see: Deng & Hu (2007 ▶). For a related structure, see: Wang *et al.* (2008 ▶).
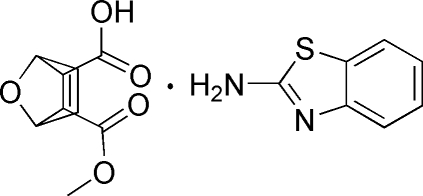


## Experimental

### 

#### Crystal data

C_7_H_6_N_2_S·C_9_H_10_O_5_
*M* *_r_* = 348.37
Monoclinic, 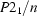

*a* = 10.2737 (10) Å
*b* = 10.4325 (11) Å
*c* = 15.0308 (17) Å
β = 93.646 (1)°
*V* = 1607.7 (3) Å^3^
*Z* = 4
Mo *K*α radiation
μ = 0.23 mm^−1^
*T* = 298 K
0.44 × 0.42 × 0.35 mm

#### Data collection

Bruker SMART CCD diffractometer
Absorption correction: multi-scan (*SADABS*; Bruker, 1997 ▶) *T*_min_ = 0.905, *T*_max_ = 0.924
7888 measured reflections
2849 independent reflections
1679 reflections with *I* > 2σ(*I*)
*R*_int_ = 0.044

#### Refinement

*R*[*F* ^2^ > 2σ(*F* ^2^)] = 0.046
*wR*(*F* ^2^) = 0.123
*S* = 1.03
2849 reflections
218 parameters
H-atom parameters constrained
Δρ_max_ = 0.20 e Å^−3^
Δρ_min_ = −0.28 e Å^−3^

### 

Data collection: *SMART* (Bruker, 1997 ▶); cell refinement: *SAINT* (Bruker, 1997 ▶); data reduction: *SAINT*; program(s) used to solve structure: *SHELXS97* (Sheldrick, 2008 ▶); program(s) used to refine structure: *SHELXL97* (Sheldrick, 2008 ▶); molecular graphics: *SHELXTL* (Sheldrick, 2008 ▶) and *PLATON* (Spek, 2009 ▶); software used to prepare material for publication: *SHELXTL*.

## Supplementary Material

Crystal structure: contains datablocks global, I. DOI: 10.1107/S1600536810052542/lh5186sup1.cif

Structure factors: contains datablocks I. DOI: 10.1107/S1600536810052542/lh5186Isup2.hkl

Additional supplementary materials:  crystallographic information; 3D view; checkCIF report

## Figures and Tables

**Table 1:** Hydrogen-bond geometry (Å, °)

*D*—H⋯*A*	*D*—H	H⋯*A*	*D*⋯*A*	*D*—H⋯*A*
N2—H2*A*⋯O3^i^	0.86	2.08	2.849 (3)	148
N2—H2*B*⋯O3^ii^	0.86	2.46	2.987 (4)	120
N2—H2*B*⋯O5^ii^	0.86	2.14	2.949 (4)	157
O4—H4⋯N1^iii^	0.82	1.89	2.676 (3)	162

Symmetry codes: (i) 
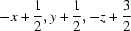
; (ii) 
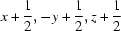
; (iii) 
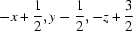
.

**Fig. 1.**
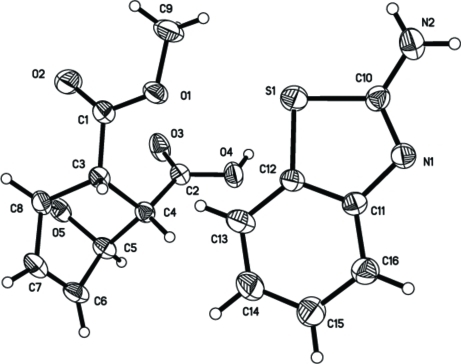
The asymmetric unit of (I), with the atom-numbering scheme. Displacement ellipsoids are drawn at 30% probability level.

**Fig. 2.**
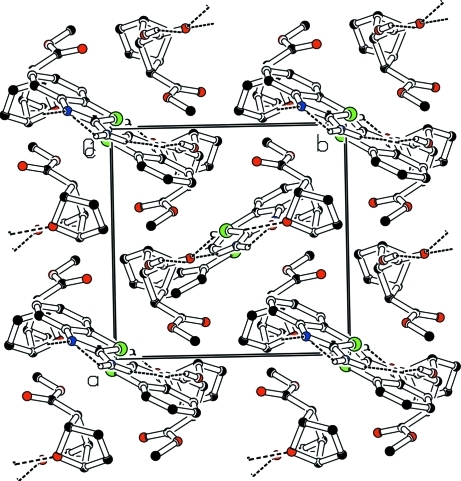
Part of the crystal structure with hydrogen bonds shown as dashed lines.

## supplementary crystallographic information

### Comment

7-Oxa-bicyclo[2,2,1]hept-5-ene-2,3-dicarboxylic anhydride has been widely employed in clinical practice (Deng & Hu, 2007). In this paper, the crystal structure of the title compound is reported. The asymmetric unit consistes of a 3-(methoxycarbonyl)-7-oxa-bicyclo[2.2.1]hept-5-ene-2-carboxylic acid molecule and a benzothiazol-2-amine molecule (Fig. 1). Bond lengths and angles are comparable to those observed for the 1:1 cocrystal of rac-7-oxabicyclo[2.2.1]heptane-2,3-dicarboxylic acid and benzothiazol-2-amine (Wang, *et al.*, 2008). In the 3-(methoxycarbonyl)-7-oxa-bicyclo[2.2.1]hept-5-ene-2-carboxylic acid molecule the dihedral angle between the mean plane formed by atoms C3/C4/C5/C8 and the plane fromed by C5/C6/C7/C8 is 69.3 (2) °. All non-hydrogen atoms of the benzothiazol-2-amine molecule are essentially co-planar with a maximum deviation of 0.0286 (9)Å for atom S1. In the crystal structure, intermolecular N—H···O and O—H···N hydrogen bonds connect two molecules of each type into centrosymmetric four component clusters (Fig. 2, Table 1).

### Experimental

A mixture of *exo*-7-oxa-bicyclo[2,2,1]hept-5-ene-2,3-dicarboxylic anhydride (0.332 g, 2 mmol) and benzothiazol-2-amine (0.3 g, 2 mmol) in methanol (5 ml) was stirred for 5 h at room temperature. The reacted solution was left for crystallization at room temperature. The single-crystal suitable for X-ray determination was obtained by evaporation after 5 d.

### Refinement

H atoms were initially located from difference maps and then refined in a riding model with C—H = 0.93–0.96 Å, N-H =0.86Å, O-H = 0.82Å  and *U*_iso_(H) = 1.2*U*_eq_(C, N) or 1.5*U*_eq_(O, methyl C).

### Crystal data

**Table:**

C_7_H_6_N_2_S·C_9_H_10_O_5_	*F*(000) = 728
*M**_r_* = 348.37	*D*_x_ = 1.439 Mg m^−^^3^
Monoclinic, *P*2_1_/*n*	Mo *K*α radiation, λ = 0.71073 Å
Hall symbol: -P 2yn	Cell parameters from 1736 reflections
*a* = 10.2737 (10) Å	θ = 2.3–22.2°
*b* = 10.4325 (11) Å	µ = 0.23 mm^−^^1^
*c* = 15.0308 (17) Å	*T* = 298 K
β = 93.646 (1)°	Block, light yellow
*V* = 1607.7 (3) Å^3^	0.44 × 0.42 × 0.35 mm
*Z* = 4	

### Data collection

**Table:**

Bruker SMART CCD diffractometer	2849 independent reflections
Radiation source: fine-focus sealed tube	1679 reflections with *I* > 2σ(*I*)
graphite	*R*_int_ = 0.044
φ and ω scans	θ_max_ = 25.0°, θ_min_ = 2.3°
Absorption correction: multi-scan (*SADABS*; Bruker, 1997)	*h* = −12→12
*T*_min_ = 0.905, *T*_max_ = 0.924	*k* = −12→11
7888 measured reflections	*l* = −17→15

### Refinement

**Table:**

Refinement on *F*^2^	Primary atom site location: structure-invariant direct methods
Least-squares matrix: full	Secondary atom site location: difference Fourier map
*R*[*F*^2^ > 2σ(*F*^2^)] = 0.046	Hydrogen site location: inferred from neighbouring sites
*wR*(*F*^2^) = 0.123	H-atom parameters constrained
*S* = 1.03	*w* = 1/[σ^2^(*F*_o_^2^) + (0.0403*P*)^2^ + 0.9023*P*] where *P* = (*F*_o_^2^ + 2*F*_c_^2^)/3
2849 reflections	(Δ/σ)_max_ < 0.001
218 parameters	Δρ_max_ = 0.20 e Å^−^^3^
0 restraints	Δρ_min_ = −0.28 e Å^−^^3^

### Special details

**Table:**

Geometry. All e.s.d.'s (except the e.s.d. in the dihedral angle between two l.s. planes) are estimated using the full covariance matrix. The cell e.s.d.'s are taken into account individually in the estimation of e.s.d.'s in distances, angles and torsion angles; correlations between e.s.d.'s in cell parameters are only used when they are defined by crystal symmetry. An approximate (isotropic) treatment of cell e.s.d.'s is used for estimating e.s.d.'s involving l.s. planes.
Refinement. Refinement of *F*^2^ against ALL reflections. The weighted *R*-factor *wR* and goodness of fit *S* are based on *F*^2^, conventional *R*-factors *R* are based on *F*, with *F* set to zero for negative *F*^2^. The threshold expression of *F*^2^ > σ(*F*^2^) is used only for calculating *R*-factors(gt) *etc*. and is not relevant to the choice of reflections for refinement. *R*-factors based on *F*^2^ are statistically about twice as large as those based on *F*, and *R*- factors based on ALL data will be even larger.

### Fractional atomic coordinates and isotropic or equivalent isotropic displacement parameters (Å^2^)

**Table:**

	*x*	*y*	*z*	*U*_iso_*/*U*_eq_	
N1	0.4153 (2)	0.6895 (2)	0.77925 (16)	0.0453 (7)	
N2	0.5207 (3)	0.5250 (3)	0.85991 (17)	0.0582 (8)	
H2A	0.5263	0.5700	0.9079	0.070*	
H2B	0.5514	0.4483	0.8601	0.070*	
O1	0.3673 (2)	0.2580 (2)	0.52228 (13)	0.0535 (6)	
O2	0.3477 (3)	0.1157 (3)	0.41181 (16)	0.0818 (9)	
O3	0.0688 (2)	0.1771 (2)	0.49890 (13)	0.0617 (7)	
O4	0.0927 (2)	0.3513 (2)	0.58303 (13)	0.0582 (6)	
H4	0.0728	0.3016	0.6222	0.087*	
O5	0.0846 (2)	0.2395 (2)	0.30892 (13)	0.0513 (6)	
S1	0.45412 (9)	0.48354 (8)	0.68714 (6)	0.0561 (3)	
C1	0.3281 (3)	0.2196 (3)	0.4405 (2)	0.0465 (8)	
C2	0.0935 (3)	0.2904 (3)	0.5075 (2)	0.0420 (8)	
C3	0.2594 (3)	0.3276 (3)	0.39140 (18)	0.0389 (7)	
H3	0.3188	0.4010	0.3896	0.047*	
C4	0.1297 (3)	0.3720 (3)	0.43033 (18)	0.0393 (7)	
H4A	0.1347	0.4625	0.4476	0.047*	
C5	0.0336 (3)	0.3534 (3)	0.3470 (2)	0.0471 (8)	
H5	−0.0588	0.3494	0.3594	0.057*	
C6	0.0675 (3)	0.4538 (3)	0.2804 (2)	0.0525 (9)	
H6	0.0209	0.5280	0.2656	0.063*	
C7	0.1755 (3)	0.4154 (3)	0.2479 (2)	0.0510 (9)	
H7	0.2214	0.4562	0.2046	0.061*	
C8	0.2104 (3)	0.2917 (3)	0.29452 (19)	0.0477 (8)	
H8	0.2683	0.2352	0.2631	0.057*	
C9	0.4275 (4)	0.1628 (3)	0.5812 (2)	0.0713 (11)	
H9A	0.3621	0.1046	0.5998	0.107*	
H9B	0.4689	0.2043	0.6325	0.107*	
H9C	0.4915	0.1162	0.5504	0.107*	
C10	0.4647 (3)	0.5736 (3)	0.7857 (2)	0.0460 (8)	
C11	0.3640 (3)	0.7130 (3)	0.6929 (2)	0.0446 (8)	
C12	0.3787 (3)	0.6131 (3)	0.6332 (2)	0.0470 (8)	
C13	0.3350 (3)	0.6251 (4)	0.5446 (2)	0.0638 (10)	
H13	0.3454	0.5582	0.5048	0.077*	
C14	0.2767 (4)	0.7362 (4)	0.5167 (2)	0.0722 (12)	
H14	0.2479	0.7453	0.4571	0.087*	
C15	0.2594 (3)	0.8357 (4)	0.5750 (2)	0.0652 (10)	
H15	0.2179	0.9102	0.5544	0.078*	
C16	0.3029 (3)	0.8262 (3)	0.6639 (2)	0.0547 (9)	
H16	0.2917	0.8936	0.7032	0.066*	

### Atomic displacement parameters (Å^2^)

**Table:**

	*U*^11^	*U*^22^	*U*^33^	*U*^12^	*U*^13^	*U*^23^
N1	0.0546 (16)	0.0395 (17)	0.0419 (16)	0.0030 (13)	0.0026 (13)	−0.0020 (12)
N2	0.085 (2)	0.0442 (18)	0.0454 (17)	0.0167 (16)	0.0036 (15)	−0.0005 (14)
O1	0.0663 (15)	0.0479 (15)	0.0437 (13)	0.0040 (11)	−0.0162 (11)	−0.0004 (11)
O2	0.122 (2)	0.0556 (17)	0.0649 (17)	0.0284 (16)	−0.0192 (15)	−0.0140 (14)
O3	0.0928 (18)	0.0521 (16)	0.0407 (13)	−0.0243 (14)	0.0078 (12)	−0.0001 (11)
O4	0.0928 (18)	0.0474 (14)	0.0354 (13)	−0.0022 (13)	0.0114 (12)	−0.0016 (11)
O5	0.0674 (15)	0.0475 (14)	0.0376 (12)	−0.0173 (12)	−0.0078 (11)	0.0021 (11)
S1	0.0723 (6)	0.0454 (6)	0.0512 (5)	−0.0042 (5)	0.0095 (4)	−0.0105 (4)
C1	0.049 (2)	0.049 (2)	0.0406 (19)	−0.0010 (17)	−0.0008 (15)	0.0009 (17)
C2	0.0448 (18)	0.045 (2)	0.0358 (19)	−0.0019 (16)	0.0019 (14)	0.0015 (16)
C3	0.0438 (18)	0.0360 (18)	0.0364 (17)	−0.0060 (14)	−0.0007 (14)	−0.0001 (14)
C4	0.0461 (18)	0.0365 (18)	0.0349 (16)	−0.0033 (15)	−0.0011 (14)	−0.0007 (14)
C5	0.0470 (19)	0.051 (2)	0.0426 (19)	−0.0059 (16)	−0.0058 (15)	0.0068 (17)
C6	0.060 (2)	0.051 (2)	0.0444 (19)	−0.0001 (18)	−0.0153 (17)	0.0096 (17)
C7	0.068 (2)	0.050 (2)	0.0335 (18)	−0.0141 (19)	−0.0058 (17)	0.0080 (16)
C8	0.061 (2)	0.049 (2)	0.0333 (17)	−0.0028 (17)	0.0037 (15)	0.0004 (15)
C9	0.085 (3)	0.065 (3)	0.060 (2)	0.011 (2)	−0.021 (2)	0.015 (2)
C10	0.0497 (19)	0.044 (2)	0.0450 (19)	−0.0030 (16)	0.0108 (15)	−0.0010 (16)
C11	0.0409 (18)	0.047 (2)	0.0455 (19)	−0.0080 (16)	0.0020 (15)	0.0001 (16)
C12	0.0486 (19)	0.052 (2)	0.0399 (19)	−0.0135 (16)	0.0028 (15)	−0.0056 (16)
C13	0.069 (2)	0.071 (3)	0.051 (2)	−0.018 (2)	−0.0037 (19)	−0.014 (2)
C14	0.071 (3)	0.093 (3)	0.050 (2)	−0.018 (2)	−0.0163 (19)	0.005 (2)
C15	0.056 (2)	0.073 (3)	0.065 (3)	−0.007 (2)	−0.0136 (19)	0.013 (2)
C16	0.054 (2)	0.053 (2)	0.056 (2)	−0.0020 (18)	−0.0012 (17)	0.0009 (18)

### Geometric parameters (Å, °)

**Table:**

N1—C10	1.312 (4)	C4—H4A	0.9800
N1—C11	1.391 (4)	C5—C6	1.504 (4)
N2—C10	1.323 (4)	C5—H5	0.9800
N2—H2A	0.8600	C6—C7	1.304 (4)
N2—H2B	0.8600	C6—H6	0.9300
O1—C1	1.330 (3)	C7—C8	1.501 (4)
O1—C9	1.443 (3)	C7—H7	0.9300
O2—C1	1.188 (4)	C8—H8	0.9800
O3—C2	1.214 (4)	C9—H9A	0.9600
O4—C2	1.302 (3)	C9—H9B	0.9600
O4—H4	0.8200	C9—H9C	0.9600
O5—C8	1.432 (4)	C11—C12	1.390 (4)
O5—C5	1.432 (4)	C11—C16	1.395 (4)
S1—C12	1.733 (3)	C12—C13	1.384 (4)
S1—C10	1.752 (3)	C13—C14	1.359 (5)
C1—C3	1.499 (4)	C13—H13	0.9300
C2—C4	1.504 (4)	C14—C15	1.377 (5)
C3—C8	1.556 (4)	C14—H14	0.9300
C3—C4	1.560 (4)	C15—C16	1.385 (4)
C3—H3	0.9800	C15—H15	0.9300
C4—C5	1.557 (4)	C16—H16	0.9300
			
C10—N1—C11	110.7 (3)	C6—C7—H7	127.1
C10—N2—H2A	120.0	C8—C7—H7	127.1
C10—N2—H2B	120.0	O5—C8—C7	101.9 (3)
H2A—N2—H2B	120.0	O5—C8—C3	101.0 (2)
C1—O1—C9	116.9 (3)	C7—C8—C3	106.5 (3)
C2—O4—H4	109.5	O5—C8—H8	115.2
C8—O5—C5	95.9 (2)	C7—C8—H8	115.2
C12—S1—C10	88.80 (16)	C3—C8—H8	115.2
O2—C1—O1	124.2 (3)	O1—C9—H9A	109.5
O2—C1—C3	126.3 (3)	O1—C9—H9B	109.5
O1—C1—C3	109.5 (3)	H9A—C9—H9B	109.5
O3—C2—O4	123.7 (3)	O1—C9—H9C	109.5
O3—C2—C4	121.9 (3)	H9A—C9—H9C	109.5
O4—C2—C4	114.3 (3)	H9B—C9—H9C	109.5
C1—C3—C8	113.2 (3)	N1—C10—N2	124.2 (3)
C1—C3—C4	115.1 (2)	N1—C10—S1	115.4 (2)
C8—C3—C4	100.9 (2)	N2—C10—S1	120.5 (3)
C1—C3—H3	109.1	C12—C11—N1	114.8 (3)
C8—C3—H3	109.1	C12—C11—C16	119.9 (3)
C4—C3—H3	109.1	N1—C11—C16	125.3 (3)
C2—C4—C5	112.0 (2)	C13—C12—C11	120.8 (3)
C2—C4—C3	112.4 (2)	C13—C12—S1	128.9 (3)
C5—C4—C3	100.0 (2)	C11—C12—S1	110.3 (2)
C2—C4—H4A	110.7	C14—C13—C12	118.9 (4)
C5—C4—H4A	110.7	C14—C13—H13	120.5
C3—C4—H4A	110.7	C12—C13—H13	120.5
O5—C5—C6	101.9 (3)	C13—C14—C15	121.2 (3)
O5—C5—C4	101.3 (2)	C13—C14—H14	119.4
C6—C5—C4	106.6 (2)	C15—C14—H14	119.4
O5—C5—H5	115.1	C14—C15—C16	120.9 (4)
C6—C5—H5	115.1	C14—C15—H15	119.5
C4—C5—H5	115.1	C16—C15—H15	119.5
C7—C6—C5	105.9 (3)	C15—C16—C11	118.2 (3)
C7—C6—H6	127.0	C15—C16—H16	120.9
C5—C6—H6	127.0	C11—C16—H16	120.9
C6—C7—C8	105.8 (3)		
			
C9—O1—C1—O2	−5.4 (5)	C6—C7—C8—O5	32.5 (3)
C9—O1—C1—C3	175.8 (3)	C6—C7—C8—C3	−73.0 (3)
O2—C1—C3—C8	1.0 (5)	C1—C3—C8—O5	87.8 (3)
O1—C1—C3—C8	179.7 (2)	C4—C3—C8—O5	−35.8 (3)
O2—C1—C3—C4	116.3 (4)	C1—C3—C8—C7	−166.1 (3)
O1—C1—C3—C4	−64.9 (3)	C4—C3—C8—C7	70.3 (3)
O3—C2—C4—C5	48.4 (4)	C11—N1—C10—N2	179.8 (3)
O4—C2—C4—C5	−132.4 (3)	C11—N1—C10—S1	0.4 (3)
O3—C2—C4—C3	−63.3 (4)	C12—S1—C10—N1	0.6 (2)
O4—C2—C4—C3	115.9 (3)	C12—S1—C10—N2	−178.8 (3)
C1—C3—C4—C2	−3.9 (3)	C10—N1—C11—C12	−1.6 (4)
C8—C3—C4—C2	118.4 (3)	C10—N1—C11—C16	179.2 (3)
C1—C3—C4—C5	−122.8 (3)	N1—C11—C12—C13	−178.4 (3)
C8—C3—C4—C5	−0.6 (3)	C16—C11—C12—C13	0.9 (5)
C8—O5—C5—C6	49.2 (2)	N1—C11—C12—S1	2.1 (3)
C8—O5—C5—C4	−60.7 (2)	C16—C11—C12—S1	−178.7 (2)
C2—C4—C5—O5	−82.4 (3)	C10—S1—C12—C13	179.0 (3)
C3—C4—C5—O5	36.8 (3)	C10—S1—C12—C11	−1.5 (2)
C2—C4—C5—C6	171.4 (3)	C11—C12—C13—C14	−0.3 (5)
C3—C4—C5—C6	−69.4 (3)	S1—C12—C13—C14	179.2 (3)
O5—C5—C6—C7	−31.4 (3)	C12—C13—C14—C15	−0.7 (6)
C4—C5—C6—C7	74.3 (3)	C13—C14—C15—C16	1.0 (6)
C5—C6—C7—C8	−0.6 (3)	C14—C15—C16—C11	−0.4 (5)
C5—O5—C8—C7	−49.7 (3)	C12—C11—C16—C15	−0.5 (5)
C5—O5—C8—C3	60.0 (2)	N1—C11—C16—C15	178.6 (3)

### Hydrogen-bond geometry (Å, °)

**Table:**

*D*—H···*A*	*D*—H	H···*A*	*D*···*A*	*D*—H···*A*
N2—H2A···O3^i^	0.86	2.08	2.849 (3)	148
N2—H2B···O3^ii^	0.86	2.46	2.987 (4)	120
N2—H2B···O5^ii^	0.86	2.14	2.949 (4)	157
O4—H4···N1^iii^	0.82	1.89	2.676 (3)	162

Symmetry codes: (i) −*x*+1/2, *y*+1/2, −*z*+3/2; (ii) *x*+1/2, −*y*+1/2, *z*+1/2; (iii) −*x*+1/2, *y*−1/2, −*z*+3/2.
